# Associations between CD160 polymorphisms and autoimmune thyroid disease: a case-control study

**DOI:** 10.1186/s12902-021-00810-w

**Published:** 2021-07-08

**Authors:** Weiwei He, Jing Zhao, Xuerong Liu, Sheli Li, Kaida Mu, Jing Zhang, Jin-an Zhang

**Affiliations:** 1 Department of Endocrinology and Rheumatology, Shanghai University of Medicine & Health Sciences Affiliated Zhoupu Hospital, Shanghai, 201508 China; 2 Department of Endocrinology, Nanjing Medical University Affiliated Wuxi People’s Hospital, Wuxi, 214000 China; 3 Department of Endocrinology, Affiliated Hospital of Yanan University, Yan’an, Shanxi China

**Keywords:** CD160, Polymorphism, Graves’ disease, Hashimoto’s thyroiditis

## Abstract

**Background:**

Recent researches suggest that the CD160/HVEM/LIGHT/BTLA signaling pathway may contribute to the pathogeneses of autoimmune diseases, but the relationship between CD160 polymorphisms and autoimmune thyroid disease (AITD) has not been reported yet. This study aimed to evaluate the associations between CD160 polymorphisms and AITD.

**Methods:**

A total of 1017 patients with AITD (634 Graves’ disease and 383 Hashimoto’s thyroiditis) and 856 unrelated healthy controls were recruited into our study. Odds ratios (ORs) with 95% confidence interval (95%CI) were calculated through logistic regression analyses. The CD160 SNPs were detected using Hi-SNP high-throughput genotyping.

**Results:**

There was a statistically significant difference between Graves’ disease patients and the control group with respect to both the genotype distribution (*P* = 0.014) and allele frequency of rs744877 (*P* = 0.034). A significant association of CD160 rs744877 with AITD was observed before adjusted age and gender under a dominant model (OR = 0.79, 95%CI 0.66–0.95; *P* = 0.013) and an additive model (OR = 0.77, 95%CI 0.64–0.94, *P* = 0.008), and was also observed after adjusted age and gender under a dominant model (OR = 0.78, 95%CI 0.65–0.95; *P* = 0.011) and an additive model (OR = 0.76, 95%CI 0.63–0.93, *P* = 0.007). A significant association of rs744877 with Graves’ disease was observed under an allele model (OR = 0.84, 95%CI 0.71–0.98, *P* = 0.027), a dominant model (OR = 0.74, 95%CI 0.60–0.91; *P* = 0.005), and an additive model (OR = 0.72, 95%CI 0.58–0.90, *P* = 0.004). Multivariate logistic regression analyses suggested that the association remained significant after adjustment for age and gender. However, rs744877 was not related to Hashimoto’s thyroiditis. Furthermore, CD160 rs3766526 was not significantly related to either Graves’ disease or Hashimoto’s thyroiditis.

**Conclusion:**

This is the first identification of the association of CD160 rs744877 with Graves’ disease. Our findings add new data to the genetic contribution to Graves’ disease susceptibility and support the crucial role of the CD160/HVEM/LIGHT/BTLA pathway in the pathogenesis of Graves’ disease.

**Supplementary Information:**

The online version contains supplementary material available at 10.1186/s12902-021-00810-w.

## Background

Autoimmune thyroid diseases (AITDs) are endocrine autoimmune diseases with a prevalence of about 5% worldwide that varies with different racial populations and in areas with different amounts of iodine intake [1–4]. AITD occurs more frequently in women than in men, and the lifetime risk of Graves’ disease (GD) is approximately 3% for women and 0.5% for men. Accounting for individuals with undiagnosed and euthyroid Hashimoto’s thyroiditis (HT), the true prevalence of HT is estimated to be about 5–10% of the general population. AITDs encompass several types, including GD, HT, atrophic autoimmune thyroiditis, postpartum thyroiditis, and Graves’ ophthalmopathy (GO) [5]. The prevalence of HT is the highest, followed by GD. Because GD and HT are the two main subtypes of AITDs and most published literature investigating the genetics of AITDs have focused on susceptibility genes of either GD or HT, we aimed to explore the susceptibility genes of GD and HT and to include only GD and HT patients in our research [6, 7]. Though its pathogenesis has not yet been clarified, it has been well accepted that genetic and environmental factors both play crucial roles in the pathogenesis of AITD, of which genetic factors contribute up to 80% of the risk factors [8–10]. Some studies have confirmed that there is a clear familial predisposition to AITD [11]. GD is characterized by a diffuse toxic goiter caused by thyroid-stimulating hormone (TSH) receptor antibodies (TRAb) and is the most common cause of hyperthyroidism [12]. TRAb binds to TSH receptors on thyroid follicle cells and stimulates the production of thyroid hormones. However, the clinical manifestations of HT are diametrically opposite, with hypothyroidism accompanied by an increase in thyroglobulin antibody (TGAb) and thyroid peroxidase antibody (TPOAb) [10]. HT, also known as chronic lymphocytic thyroiditis or autoimmune thyroiditis, is the most common type of AITD. It is also the most common organ-specific autoimmune disease and the most common cause of hypothyroidism. The pathological feature of HT is the local infiltration of lymphocytes, accompanied by elevated levels of TGAb and TPOAb. Environmental factors affecting the occurrence of HT mainly include high iodine intake, infection, stress, and radiation exposure [7, 13]. There is evidence that many of the susceptibility genes are shared by various autoimmune diseases, such as CTLA4 and PTPN22, which indicate that autoimmune diseases have some similar genetic and molecular pathways [14, 15]. Previous studies have reported that there are many susceptibility genes associated with AITD. Generally, most of those susceptibility genes can be classified into two types. One is thyroid-associated genes including TSHR and TG, and the other is immunity-related genes, such as FOXP3 [16], HLA-DR genes [17], and CD40 [15]. Some other novel AITD genes have also been reported, such as VDR [18, 19] and FCRL3 [20].

Herpesvirus entry mediator (HVEM) is a receptor on immune cells, which is also known as tumor necrosis factor receptor superfamily member 14 (TNFRSF14) [21]. CD160, B- and T-lymphocyte attenuating agents (BTLA), lymphotoxin-like (LIGHT), and lymphotoxin-alpha (LTa) are ligands for HVEM [22, 23]. The CD160/HVEM/LIGHT/BTLA pathway is an essential signaling pathway in regulating immunity, which is involved in the pathogeneses of infection and autoimmunity [24–26].. Some gene polymorphisms in the CD160/HVEM/LIGHT/BTLA pathway have an obvious influence on their functions and are thus associated with host susceptibility to autoimmune diseases [27–31].. The inhibitory co-stimulator CD160, originally named BY55, is an immunoglobulin-like activator of the NK cell receptor and belongs to the glycosylphosphatidylinositol (GPI) fixed membrane protein family. The gene is located at 1q42.3 [32, 33]. There are also several single nucleotide polymorphisms (SNPs) in CD160. A study found that CD160 rs744877 was closely related to the susceptibility of rheumatoid arthritis (RA) and may be involved in the development of RA [34]. rs3766526 of CD160 was identified with data from the 1000 Genomes Project (http://phase3browser.1000genomes.org/index.html), and it met the selection criteria of MAF more than 0.10 and r^2^ more than 0.8 in the linkage disequilibrium analysis. Currently, there is no study on the relationship between CD160 genetic polymorphisms and AITD, and thus the role of CD160 genetic polymorphisms in the susceptibility to AITD remains unclear. Herein, we report a case-control involving a large sample to investigate the relationship between two CD160 SNPs (rs744877 and rs3766526) and AITD in a Chinese Han population.

## Methods

### Study subjects

The study group consisted of 1017 patients with AITD (634 GD and 383 HT) and 856 healthy controls. AITD patients were recruited from the Department of Endocrinology, Jinshan Hospital of Fudan University. Both GD and HT are well-characterized autoimmune thyroid disorders with a variety of clinical manifestations. The diagnostic hallmark of GD or HT is the presence of circulating autoantibodies. TGAb and TPOAb can be found in most HT patients. Some HT patients with serious thyroid damage caused by immune system activity and lymphocytic infiltration may have hypothyroidism symptoms, but hypothyroidism is usually absent in a large portion of HT patients and these patients are diagnosed with HT with euthyroidism in clinical practice. HT patients may present with various states of thyroid function, but most will eventually evolve into hypothyroidism [13]. Briefly, all AITD patients were diagnosed based on clinical manifestations and laboratory tests. GD patients were diagnosed by the presence of hyperthyroidism, a toxic diffuse goiter, and a positive test for TRAb. HT was mainly diagnosed by TGAb positivity or TPOAb positivity and diffuse hypoechogenicity on thyroid ultrasound, with or without hypothyroidism. AITD patients with other autoimmune diseases as well as genetic diseases were excluded. By asking about their disease history and family history, we excluded those individuals with other autoimmune or genetic diseases and included individuals with AITD alone. Healthy controls were matched by geographic region. Controls with the presence of thyroid disease or other autoimmune disorders and those with severe or chronic inflammatory diseases were excluded. The diagnosis of GO is confirmed by the presence of GD diagnosis and the associated ocular symptoms. This study was approved by the ethics committee of Jinshan Hospital, and written informed consent was obtained from all participants.

### Genotyping

2 ml of peripheral blood was obtained by venipuncture and collected in an EDTA tube. Extraction of genomic DNA from peripheral blood leukocytes using the standard procedure of the RelaxGene Blood DNA System (Tiangen Biotech, Beijing, China). Moreover, In addition, DNA sample concentrations and A260 / A280 ratios were measured using the Nano Drop 2000 spectrophotometer (Thermo Scientific Company, Waltham, MA, USA) [35, 36]. The CD160 SNPs were genotyped using a high throughput-SNP (Hi-SNP) genotyping method with technical support from the Shanghai Biowing Applied Biotechnology company, which was based on three-round multiplex PCR coupled with next-generation sequencing. The details of the Hi-SNP genotyping method have been reported in previously published studies [37, 38]. Briefly, DNA was first amplified in a 10ul PCR reaction using the following PCR thermocycling conditions: 95 °C for 15 min (1 cycle); 94 °C for the 30s, 60 °C for 10 min and 72 °C for 30s (4 cycles); 94 °C for 30s, 60 °C for 1 min and 72 °C for 30s (20 cycles). PCR products in the first round were then amplified for the second PCR, and PCR products from the second round were further amplified under PCR thermocycling conditions used in previous studies [37, 38]. The SNPs were directly identified by mapping targeted sequences to the SNP reference sequences (NCBI, dbSNP build 142) using BWA (v0.7.12) and samtools (v0.1.19). The forward and reverse primers in the amplification for rs744877 were 5′-GCTGACCTTCCAGCTCCTCTG-3′ and 5′-CTGGACCTCACTCAGCCTCAC-3, respectively. The forward and reverse primers in the amplification for rs3766526 were 5′-ATATACTCAATCTGGCCATCAGC-3′ and 5′-TTTTCAATGGGTAGGAGGTAAGAC-3′, respectively.

### Statistical analysis

STATA was used for the statistical analysis. Hardy-Weinberg equilibrium (HWE) was evaluated using the χ2 test. A Chi-square test was used to compare allele and genotype frequencies between cases and controls. Through the dominant model, allelic model, homozygous model, recessive model, and additive model, the relationship between CD160 and AITD including GD and HT was analyzed. Multiple logistic regression analysis was also performed, and age and gender were used as confounding factors. P < 0.05 was considered significant. Before starting our research, we had done a power analysis to calculate the sample size with an online power and sample size calculation software (http://www.openepi.com/Menu/OE_Menu.htm). We calculated the power for rs3766526 because the rs3766526 MAF of Chinese in 1000 Genomes Project was lower than that of rs744877 (18.5% vs 30.3%) and a larger sample size was needed to find a statistically significant association. When the expected OR defined as 1.35, a sufficient power of over 80% required a combined set of 530 cases and 530 controls. Moreover, 1017 AITD cases and 856 controls in our study had a power of 99.6% in discovering a significant difference between AITD cases and controls. Six hundred thirty-four GD cases and 856 controls had a power of 98.7% in finding an obvious difference between GD cases and controls. six hundred thirty-four HT cases and 856 controls had a power of 94.8% in finding an obvious difference between HT cases and controls. Therefore, the sample size in our study was suitably powered.

## Results

### Subject features

The baseline data for this study is shown in Table 1. There were 634, 383, and 856 individuals in the GD, HT, and control groups, respectively. In the GD group, 446 (70.35%) individuals were females and 188 (29.65%) were males. Among them, 135 (21.29%) individuals had a positive family history of AITD and 100 (15.77%) had ophthalmopathy. There were 171 (26.97%) patients with an I degree goiter, 293 (46.21%) patients with a II degree goiter, and 43 (6.79%) patients with a III degree goiter. In the HT group, 328 (85.64%) individuals were females and 55 (14.36%) were males. 61 (15.93%) individuals in the HT group had a positive family history of AITD. Similarly, there were 92 (24.02%) patients with an I degree goiter, 128 (33.42%) patients with a II degree goiter, and 8 (2.09%) patients with a III degree goiter in the HT group. The average age in the GD group and HT group was 41.57 ± 14.47 years and 41.59 ± 14.43 years, respectively. Among the 856 healthy controls, 499 (58.29%) individuals were females and 357 (41.71%) were males. Age and gender were different between patient and control groups (*P* < 0.05). No deviation from the HWE was observed for both rs744877 and rs3766526 in the controls (*P* > 0.05).

**Table 1:** Baseline clinical and demographics features of the study patients

Items	GD	HT	Controls
Number	634	383	856
**Gender**			
Male	188 (29.65%)	55 (14.36%)	357 (41.71%)
Female	446 (70.35%)	328 (85.64%)	499 (58.29%)
Age (years)	41.57 ± 14.47	41.59 ± 14.43	39.30 ± 9.71
**Goiter**			
No goiter	127 (20.03%)	155 (40.47%)	–
Degree I	171 (26.97%)	92 (24.02%)	–
Degree II	293 (46.21%)	128 (33.42%)	–
Degree III	43 (6.79%)	8 (2.09%)	–
**Family history**			
(+)	135 (21.29%)	61 (15.93%)	–
(−)	499 (78.71%)	322 (84.07%)	–
**Ophthalmopathy**			
(+)	100 (15.77%)	3 (0.78%)	–
(−)	534 (84.23%)	380 (99.22%)	–

*GD* Graves’ disease, *HT* Hashimoto’s thyroiditis

### Association between rs744877 and AITD

The genotyping results of all patients and controls are shown in Table 2. In AITD subjects, the genotype frequencies of AA, AC, and CC in rs744877 polymorphism were 47.89, 41.79, and 10.32%, respectively, whereas those in the control group were 42.17, 47.66, and 10.17%, respectively (Table 2). In GD subjects, the genotype frequencies of AA, AC, and CC in the rs744877 polymorphism were 49.53, 40.54, and 9.93%, respectively. In HT subjects, the genotype frequencies of AA, AC, and CC in the rs744877 locus were 45.17, 43.86, and 10.97%, respectively. Our result showed that there was a statistically significant difference between AITD patients and the control group with respect to the genotype distribution of rs744877 (*P* = 0.031). Obvious differences for both the genotype distribution (*P* = 0.014) and allele frequencies of rs744877 (*P* = 0.034) were observed between GD patients and controls. However, there was no difference between HT patients and the control group with respect to both the genotype distribution (*P* = 0.464) and allele frequencies of rs744877 (*P* = 0.593).

**Table 2:** Allele frequencies and genotype distribution of CD160 polymorphisms in AITD patients and controls

Gene/SNP	Controls	AITD	***P*** value	GD	***P***-Value	HT	***P*** value
CD160	n (%)	n (%)	AITD vs. Control	n (%)	GD vs. Control	n (%)	HT vs. Control
rs744877							
A	1130 (66%)	1399 (68.78%)	0.071	885 (69.69%)	0.034	514 (67.10%)	0.593
C	582 (34%)	635 (31.22%)		385 (30.31%)		252 (32.90%)	
AA	361 (42.17%)	487 (47.89%)	0.031	314 (49.53%)	0.014	173 (45.17%)	0.464
AC	408 (47.66%)	425 (41.79%)		257 (40.54%)		168 (43.86%)	
CC	87 (10.17%)	105 (10.32%)		63 (9.93%)		42 (10.97%)	
rs3766526							
G	1406 (82.13%)	1636 (80.43%)	0.186	1013 (79.89%)	0.123	623 (81.33%)	0.635
A	306 (17.87%)	398 (19.57%)		255 (20.11%)		143 (18.67%)	
GG	574 (67.06%)	659 (64.80%)	0.319	407 (64.20%)	0.187	252 (65.80%)	0.888
GA	258 (30.14%)	318 (31.27%)		199 (31.39%)		119 (31.07%)	
AA	24 (2.80%)	40 (3.93%)		28 (4.41%)		12 (3.13%)	

*AITD* autoimmune diseases, *GD* Graves’ disease, *HT* Hashimoto’s thyroiditis

For rs744877, the allele model was for C vs A, the dominant model was for CC/AC vs AA, the recessive model was for CC vs AA/AC, the homozygous model was for CC vs AA, and the additive model was for AC vs AA. As shown in Table 3, a significant association of CD160 rs744877 with AITD was observed under both the dominant model (OR = 0.79, 95%CI 0.66–0.95, *P* = 0.013) and additive model (OR = 0.77, 95%CI 0.64–0.94; *P* = 0.008). Multivariate logistic regression analysis suggested that the association remained significant after adjusting for age and gender (Table 3).

**Table 3:** Associations of two polymorphisms in the CD160 gene with AITD before and after adjusting for confounders

Comparison models	Unadjusted estimates		Adjusted estimates^**a**^	
	OR (95%CI)	***P*** values	OR (95%CI)	***P*** values
**rs744877**				
Allele Model	0.88 (0.77–1.01)	0.069	0.88 (0.76–1.01)	0.066
Dominant Model	0.79 (0.66–0.95)	0.013	0.78 (0.65–0.95)	0.011
Recessive model	1.02 (0.75–1.37)	0.909	1.03 (0.76–1.40)	0.864
Homozygous model	0.89 (0.65–1.23)	0.489	0.90 (0.65–1.24)	0.528
Additive model	0.77 (0.64–0.94)	0.008	0.76 (0.63–0.93)	0.007
**rs3766526**				
Allele Model	1.12 (0.95–1.32)	0.185	1.12 (0.94–1.32)	0.197
Dominant Model	1.11 (0.91–1.34)	0.305	1.10 (0.90–1.34)	0.354
Recessive model	1.42 (0.85–2.37)	0.182	1.49 (0.88–2.52)	0.138
Homozygous model	1.45 (0.86–2.44)	0.159	1.51 (0.89–2.57)	0.126
Additive model	1.07 (0.88–1.31)	0.484	1.06 (0.86–1.30)	0.579

*AITD* autoimmune diseases, *OR* Odds ratio, *95%CI* 95% confidence interval; ^a^ Age and gender were adjusted in the multivariate logistic regression analyses)

As shown in Table 4, a significant association of CD160 rs744877 with GD was observed under the allele model (OR = 0.84, 95%CI 0.71–0.98, *P* = 0.027), dominant model (OR = 0.74, 95%CI 0.60–0.91; *P* = 0.005), and additive model (OR = 0.72, 95%CI 0.58–0.90, *P* = 0.004), Multivariate logistic regression analyses suggested that the association was significant after adjusting for age and gender (Table 4).

**Table 4:** Associations of two polymorphism models in the CD160 gene with GD before and after adjusting for confounders

Comparison models	Unadjusted estimates		Adjusted estimates^**a**^	
	OR (95%CI)	***P*** values	OR (95%CI)	***P*** values
**rs744877**				
Allele Model	0.84 (0.71–0.98)	0.027	0.84 (0.71–0.98)	0.030
Dominant Model	0.74 (0.60–0.91)	0.005	0.74 (0.60–0.92)	0.005
Recessive model	0.98 (0.69–1.37)	0.886	0.98 (0.70–1.39)	0.923
Homozygous model	0.83 (0.58–1.19)	0.315	0.84 (0.58–1.20)	0.339
Additive model	0.72 (0.58–0.90)	0.004	0.72 (0.58–0.90)	0.004
**rs3766526**				
Allele Model	1.16 (0.96–1.39)	0.122	1.14 (0.95–1.38)	0.160
Dominant Model	1.14 (0.91–1.41)	0.250	1.12 (0.90–1.40)	0.295
Recessive model	1.60 (0.92–2.79)	0.096	1.54 (0.88–2.70)	0.130
Homozygous model	1.65 (0.94–2.88)	0.081	1.57 (0.89–2.77)	0.116
Additive model	1.09 (0.87–1.36)	0.462	1.08 (0.86–1.36)	0.502

*GD* Graves’ disease, *OR* Odds ratio, *95%CI* 95% confidence interval; ^a^ Age and gender were adjusted in the multivariate logistic regression analyses)

As shown in Table 5, no significant association of CD160 rs744877 with HT was observed under all models (Table 5, *P* > 0.05). Besides, no association of rs744877 with HT was observed after adjustment for age and gender (Table 5).

**Table 5:** Associations of two polymorphism models in the CD160 gene with HT before and after adjusting for confounders

Comparison models	Unadjusted estimates		Adjusted estimates^**a**^	
	OR (95%CI)	***P*** values	OR (95%CI)	***P*** values
**rs744877**				
Allele Model	0.95 (0.79–1.14)	0.585	0.95 (0.79–1.16)	0.632
Dominant Model	1.06 (0.82–1.37)	0.664	1.07 (0.82–1.40)	0.615
Recessive model	1.09 (0.74–1.61)	0.669	1.11 (0.73–1.67)	0.629
Homozygous model	1.01 (0.67–1.52)	0.972	1.04 (0.68–1.60)	0.860
Additive model	0.86 (0.67–1.11)	0.244	0.86 (0.66–1.12)	0.262
**rs3766526**				
Allele Model	1.06 (0.85–1.32)	0.631	1.09 (0.86–1.39)	0.457
Dominant Model	0.89 (0.69–1.13)	0.325	0.89 (0.69–1.14)	0.354
Recessive model	1.12 (0.55–2.27)	0.750	1.46 (0.69–3.09)	0.322
Homozygous model	1.14 (0.56–2.31)	0.719	1.45 (0.69–3.08)	0.328
Additive model	1.05 (0.81–1.37)	0.713	1.04 (0.79–1.37)	0.785

*HT* Hashimoto’s thyroiditis, *OR* Odds ratio, *95%CI* 95% confidence interval; ^a^ Age and gender were adjusted in the multivariate logistic regression analyses

Subgroup analysis of patients with GD ophthalmopathy showed a significant difference in the genotype distribution of rs744877 between patients without ophthalmopathy and controls (*P* = 0.018; Supplementary Table 1). Subgroup analysis by gender revealed that rs744877 was significantly associated with GD in women, but minimally associated with GD in men, in terms of genotype distribution (*P* < 0.001) and allele frequency (*P* = 0.004) (Supplementary Tables 2 and 3). The results of subgroup analyses stratified by family history and degree of goiter are presented in the accompanying tables (Supplementary Tables 4, 5, 6 and 7).

### Association between rs3766526 and AITD

As shown in Table 2, there was no difference between the patient group and the control group with respect to both the genotype distribution (*P* = 0.319) and allele frequency of CD160 rs3766526 (*P* = 0.186). No difference for both the genotype distribution (*P* = 0.187) and allele frequencies of rs3766526 (*P* = 0.123) was observed between GD patients and controls. There was no significant finding between HT cases and controls in both the genotype distribution (*P* = 0.888) and allele frequencies of rs3766526 (*P* = 0.635).

For rs3766526, the allele model was for A vs G, the dominant model was for AA/GA vs GG, the recessive model was for AA vs GG/GA, the homozygous model was for AA vs GG, and the additive model was for GA vs GG. As shown in Table 3, no significant association of CD160 rs3766526 with AITD was observed under all comparison models (Table 3, *P* > 0.05). Multivariate logistic regression analyses suggested that the association was not yet significant after adjustment for age and gender (Table 3). CD160 rs3766526 was also not related to either GD or HT under all comparison models (Tables 4 and 5, *P* > 0.05).

Although neither genotype distribution nor allele frequency of rs3766526 differed between GD patients and controls (Table 2), it is noteworthy that the allele frequency of rs3766526 differed significantly between GO patients and controls (*P* = 0.039; Supplementary Table 1). Subgroup analysis by sex revealed that rs3766526 was not associated with GD and HT in females, but was significantly associated with HT in males, both in terms of genotype distribution (*P* < 0.001) and allele frequency (*P* = 0.015) (Supplementary Table 3), suggesting that rs3766526 may be associated with the onset of HT. CD160 rs3766526 is a variant in 3′-UTR which often contains regulatory regions for post-transcriptional modification. Therefore, rs3766526 may have an impact on the expression of CD160 by influencing post-transcriptional modification, and thus a change in the function of the CD160/HVEM/LIGHT/BTLA pathway. Outcomes of the subgroup analyses stratified by family history and goiter degree for rs3766526 are shown in the supplementary tables (Supplementary Tables 4, 5, 6 and 7).

## Discussion

In this study, we explored two SNPs in CD160 and found an obvious relationship between CD160 rs744877 and GD. This correlation was also confirmed through multivariate logistic regression analysis. However, our study did not find any correlation between rs744877 and HT. To our knowledge, it is the first study on the linkage of CD160 polymorphisms to AITD.

The CD160/HVEM/LIGHT/BTLA pathway is an essential signaling pathway in regulating immunity, which is involved in the pathogeneses of infection and autoimmunity [22, 23, 39]. CD160, BTLA, LIGHT, and LTa all are ligands for HVEM, but they exert different regulatory roles [22, 23]. The binding of LIGHT or LTa to HVEM stimulates costimulatory signals, but the binding of BTLA or CD160 to HVEM initiates co-suppression signals, and thus the CD160/HVEM/LIGHT/BTLA costimulatory/ co-suppression pathway is a bidirectional switch that adjusts T cell activation and takes an important part in regulating immune responses [24, 40, 41]. Although the binding of LIGHT or LTa to HVEM promotes T cell activation, HVEM predominantly shows negative regulatory effects mediated by BTLA and CD160 [22, 39]. The cysteine concentration 1 (CRD1) region in HVEM molecule is responsible for the combination of CD160 and BTLA, and the deletion of HVEM CRD1 region could block the combination of CD160/BTLA and HVEM and eliminate the inhibitory effect. Thus, HVEM can be transformed into a dominant co-stimulator and enhance the immune response, even causing graft rejection [42]. Some observational studies have revealed that these genes are aberrantly expressed in patients with autoimmune diseases [43–45]. In addition, animals with knock-out of those members in the CD160/HVEM/LIGHT/BTLA signaling pathway could change the development or progression of autoimmune diseases, such as colitis and atopic dermatitis [46, 47].

Recent genetic studies have identified an association of CD160/HVEM/LIGHT/BTLA pathway-related genes with autoimmune diseases [48]. HVEM (TNFRSF14) rs6684865 was related to primary sclerosing cholangitis (PSC), RA, and multiple sclerosis (MS) [30, 48, 49]. The findings from our study prove that CD160 rs744877 is related to GD, which adds new evidence for the roles of the CD160/HVEM/LIGHT/BTLA pathway in autoimmune diseases. CD160 is an essential member of the CD160/HVEM/LIGHT/BTLA pathway, and its role in immunity has been widely recognized [50]. CD160 is highly expressed in the spleen, small intestine, and peripheral blood, but is lowly expressed in the brain, liver, heart, thymus, and other tissues [25, 32, 33]. In the immune system, CD160 is a costimulatory receptor molecule and is expressed on multiple immune cells, including intestinal intraepithelial T lymphocytes, CD56dimCD16+ NK lymphocytes, and a minor subset of CD4+ T cells and CD8+ T cells [51].. In the pathogeneses of GD, antigen-presenting cells (APC), T cells, and B cells are all involved, and the dysfunction of one type of immune cells can further lead to aberrant function of other immune cells [10]. CD160 primarily functioning as a costimulatory receptor molecule. CD160 is an essential member of the CD160/HVEM/LIGHT/BTLA pathway, and its role in immunity has been widely recognized. Therefore, CD160 plays an important role in the maintenance of immune tolerance and the prevention of autoimmune diseases. More importantly, one of our recent study suggested that CD160 was differentially expressed on the surface of CD8^+^ T cells between GD patients and healthy controls (*P* = 0.002), which suggested that CD160 dysfunction was involved in the pathogenesis of GD [52]. CD160 SNP may lead to impairment of its immunomodulatory function by affecting its expression or function. Therefore, aberrant expression or function of CD160 can first result in the dysfunction of T cells, which may further cause the activation of antibody-producing B lymphocytes and lead to the development of GD. CD160 has a higher affinity for HVEM and can inhibit CD4^+^ T cell proliferation and cytokine secretion after cross-linking [51]. Therefore, CD160 plays an important role in the maintenance of immune tolerance and the prevention of autoimmune diseases [25, 53].

Though our study indicated CD160 rs744877 locus is a susceptible factor for GD, the molecular mechanisms underlying the roles of CD160 rs744877 in autoimmune diseases are still unclear. CD160 rs744877 is an intronic SNP and is unable to act by changing the composition of amino acids or the protein structure of CD160. One possibility is that CD160 rs744877 takes part in disease development by impacting its expression or post-translational modification. CD160 polymorphisms may not be related to HT due to the different molecular mechanisms between GD and HT. Though both GD and HT are similar autoimmune diseases occurring in the same organ, they still may have some distinct molecular mechanisms [8, 54]. Previous studies also suggested several genes are differently associated with GD and HT, such as BTG1 and TSHR. Therefore, the associations of CD160 polymorphisms with GD and HT may be different, as CD160 may exert different roles in the pathogeneses of GD and HT. Another explanation was the limited number of HT patients in our study, which may cause low statistical power to detect a statistically significant relationship. Finally, subgroup analyses by gender found that rs3766526 was not associated with GD and HT in females, but it was significantly associated with HT in males for both the genotype distribution (*P* < 0.001) and allele frequencies (*P* = 0.015) (Supplementary Table 3), which suggested that the gender-specific association of rs3766526 with HT was possible. However, the finding above may be biased by the limited sample size in the subgroup analyses by gender, and studies with larger sample sizes are recommended to provide a more precise evaluation of the gender-specific association of rs3766526 with HT. Similar to other published gene-association studies, our study did not perform a replication study, which was due to the limited number of AITD DNA samples in our clinical biobank of AITD. To ensure the reliability of the findings in our study, we attempted to collect more AITD samples, and the sample size in our study is relatively large compared with other published case-control studies on the susceptibility genes of AITD. Moreover, the sample size calculation also suggested that the sample size in our study was large enough to assess associations adequately. Our study focused on the relationship between AITD and controls, and from this perspective the number of patients was sufficient to assess the relationship between CD160 SNPs and AITD. However, when subgroup analysis was done, there were fewer HT patients, which may lead to low statistical power in assessing the relationship between CD160 SNPs and HT. Nevertheless, the findings in our study still need to be validated in future studies, especially for those from other countries or populations.

## Conclusion

In conclusion, this is the first demonstration of the association of CD160 rs744877 with GD and supports the critical role of the CD160 / HVEM / LIGHT / BTLA pathway in the pathogenesis of GD.

## Supplementary Information

**Additional file 1: Supplementary Table 1.** Allele frequencies and genotype distribution of CD160 polymorphisms between GD patients and controls in the subgroup analysis by ophthalmopathy. **Supplementary Table 2.** Allele frequencies and genotype distribution of CD160 polymorphisms between AITD patients and controls in females. **Supplementary Table 3.** Allele frequencies and genotype distribution of CD160 polymorphisms between AITD patients and controls in males. **Supplementary Table 4.** Allele frequencies and genotype distribution of CD160 polymorphisms between AITD patients and controls stratified by family history. **Supplementary Table 5.** Allele frequencies and genotype distribution of CD160 polymorphisms between GD patients and controls stratified by family history. **Supplementary Table 6.** Allele frequencies and genotype distribution of CD160 polymorphisms between HT patients and controls stratified by family history. **Supplementary Table 7.** Allele frequencies and genotype distribution of CD160 polymorphisms between AITD patients and controls stratified by goiter.

## Data Availability

Data sharing is not applicable to this article, as no datasets were generated or analyzed during the current study.

## Abbreviations

AITDs: Autoimmune thyroid diseases
GD: Graves’ disease
HT: Hashimoto’s thyroiditis
GO: Graves’ ophthalmopathy
TSH: Thyroid-stimulating hormone receptor antibodies (TRAb)
TRAb: Thyroid-stimulating hormone receptor antibodies
TGAb: Thyroglobulin antibody
TPOAb: Thyroid peroxidase antibody
HVEM: Herpesvirus entry mediator
TNFRSF14: Tumor necrosis factor receptor superfamily member 14
BTLA: B- and T-lymphocyte attenuating agents
SNPs: Single nucleotide polymorphisms
RA: Rheumatoid arthritis
Hi-SNP: High throughput-SNP
PSC: Primary sclerosing cholangitis
MS: Multiple sclerosis
APC: Antigen-presenting cells
