# Supplementary material for: Associations between CD160 polymorphisms and autoimmune thyroid disease: a case-control study
Source: BMC Endocr Disord. 2021 Jul 8;21:148. doi: 10.1186/s12902-021-00810-w (PMC8268507; doi:10.1186/s12902-021-00810-w)
Supplement: Supplementary file 1 — Additional file 1: Supplementary Table 1. Allele frequencies and genotype distribution of CD160 polymorphisms between GD patients and controls in the subgroup analysis by ophthalmopathy. Supplementary Table 2. Allele frequencies and genotype distribution of CD160 polymorphisms between AITD patients and controls in females. Supplementary Table 3. Allele frequencies and genotype distribution of CD160 polymorphisms between AITD patients and controls in males. Supplementary Table 4. Allele frequencies and genotype distribution of CD160 polymorphisms between AITD patients and controls stratified by family history. Supplementary Table 5. Allele frequencies and genotype distribution of CD160 polymorphisms between GD patients and controls stratified by family history. Supplementary Table 6. Allele frequencies and genotype distribution of CD160 polymorphisms between HT patients and controls stratified by family history. Supplementary Table 7. Allele frequencies and genotype distribution of CD160 polymorphisms between AITD patients and controls stratified by goiter. [file 12902_2021_810_MOESM1_ESM.doc]

Supplementary table 1 Allele frequencies and genotype distribution of CD160 polymorphisms between GD patients and controls in the subgroup analysis by ophthalmopathy

| **SNP** | **Allele/**  **Genotypes** | **Controls (%)** | **GO (%)** | **P^#^** | **GD without GO (%)** | **P^&^** | **P^*^** | **P^§^** |
| --- | --- | --- | --- | --- | --- | --- | --- | --- |
| rs744877 | A | 1130  (66.0%) | 145  (72.5%) | 0.065 | 740  (69.3%) | 0.073 | 0.364 | 0.062 |
|  | C | 582  (34.0%) | 55  (27.5%) |  | 328  (30.7%) |  |  |  |
|  | AA | 361  (42.2%) | 51  (51.0%) | 0.163 | 263  (49.2%) | 0.018 | 0.353 | 0.032 |
|  | AC | 408  (47.7%) | 43  (43.0%) |  | 214  (40.1%) |  |  |  |
|  | CC | 87  (10.1%) | 6  (6.0%) |  | 57  (10.7%) |  |  |  |
| rs3766526 | G | 1406  (82.1%) | 131  (75.7%) | 0.039 | 855  (80.1%) | 0.173 | 0.191 | 0.076 |
|  | A | 306  (17.9%) | 42  (24.3%) |  | 213  (19.9%) |  |  |  |
|  | GG | 574  (67.1%) | 63  (63.0%) | 0.417 | 344  (64.4%) | 0.262 | 0.936 | 0.477 |
|  | GA | 258  (30.1%) | 32  (32.0%) |  | 167  (31.3%) |  |  |  |
|  | AA | 24  (2.8%) | 5  (5.0%) |  | 23  (4.3%) |  |  |  |

(**^§^** P value for the compareiosn of three groups; **^#^** P value for GO patients versus Controls; **^&^** P value for Non-ophthalmopathy GD patients versus Controls; **^*^** P value for GO patients versus Non-ophthalmopathy GD patients. GD, Graves' disease; GO, Graves' ophthalmopathy)

**Supplementary table 2 Allele frequencies and genotype distribution of CD160** polymorphisms between AITD patients and controls in females

| **Gene/SNP** | **Controls** | **AITD** | | **P value^#^** | **GD** | **P value^&^** | **HT** | **P value^*^** |
| --- | --- | --- | --- | --- | --- | --- | --- | --- |
| rs744877 |  | | | | | | | |
| A | 663  (66.4%) | | 1059  (68.4%) | 0.298 | 548  (72.9%) | 0.004 | 441  (67.2%) | 0.738 |
| C | 335  (33.6%) | | 489  (31.6%) |  | 204  (27.1%) |  | 215  (32.8%) |  |
| AA | 211  (42.3%) | | 364  (47.0%) | 0.152 | 215  (57.2%) | <0.001 | 149  (45.4%) | 0.393 |
| AC | 241  (48.3%) | | 331  (42.8%) |  | 118  (31.4%) |  | 143  (44.6%) |  |
| CC | 47  (9.4%) | | 79  (10.2%) |  | 43  (11.4%) |  | 36  (10.0%) |  |
| rs3766526 |  | | | | | | | |
| G | 817  (81.9%) | | 1249  (80.7%) | 0.458 | 706  (79.2%) | 0.136 | 543  (82.8%) | 0.636 |
| A | 181  (18.1%) | | 299  (19.3%) |  | 186  (20.8%) |  | 113  (17.2%) |  |
| GG | 332  (66.5%) | | 503  (64.0%) | 0.681 | 284  (63.7%) | 0.123 | 219  (66.8%) | 0.300 |
| GA | 153  (30.7%) | | 243  (32.4%) |  | 138  (30.9%) |  | 105  (32.0%) |  |
| AA | 14  (2.8%) | | 28  (3.6%) |  | 24  (5.4%) |  | 4  (1.2%) |  |

(# P value for AITD patients versus Controls; & P value for GD patient versus Controls; * P value for HT patients versus Controls. AITD, autoimmune diseases; GD, Graves' disease; HT, Hashimoto's thyroiditis)

**Supplementary table 3 Allele frequencies and genotype distribution of CD160** polymorphisms between AITD patients and controls in males

| **Gene/SNP** | **Controls** | **AITD** | **P value^#^** | **GD** | **P value^&^** | **HT** | **P value^*^** |
| --- | --- | --- | --- | --- | --- | --- | --- |
| rs744877 |  | | | | | | |
| A | 467  (65.4%) | 340  (69.0%) | 0.099 | 267  (70.0%) | 0.061 | 73  (66.4%) | 0.844 |
| C | 247  (34.6%) | 146  (31.0%) |  | 109  (29.0%) |  | 37  (33.6%) |  |
| AA | 150  (42.0%) | 123  (50.6%) | 0.102 | 99  (52.7%) | 0.050 | 24  (43.6%) | 0.975 |
| AC | 167  (46.8%) | 94  (38.7%) |  | 69  (36.7%) |  | 25  (45.5%) |  |
| CC | 40  (11.2%) | 26  (10.7%) |  | 20  (10.6%) |  | 6  (10.9%) |  |
| rs3766526 |  | | | | | | |
| G | 589  (82.5%) | 387  (79.6%) | 0.211 | 307  (81.7%) | 0.729 | 80  (72.7%) | 0.015 |
| A | 125  (17.5%) | 99  (20.4%) |  | 69  (18.3%) |  | 30  (27.3%) |  |
| GG | 242  (67.8%) | 156  (64.2%) | 0.336 | 123  (65.4%) | 0.707 | 33  (60.0%) | <0.001 |
| GA | 105  (29.4%) | 75  (30.9%) |  | 61  (32.4%) |  | 14  (25.5%) |  |
| AA | 10  (2.8%) | 12  (4.9%) |  | 4  (2.2%) |  | 8  (14.5%) |  |

(# P value for AITD patients versus Controls; & P value for GD patient versus Controls; * P value for HT patients versus Controls. AITD, autoimmune diseases; GD, Graves' disease; HT, Hashimoto's thyroiditis)

Supplementary table 4 Allele frequencies and genotype distribution of CD160 polymorphisms between AITD patients and controls stratified by family history

| **SNP** | **Allele/**  **Genotypes** | **Controls (%)** | **AITD with family history (%)** | **P^#^** | **AITD without family history (%)** | **P^&^** | **P^*^** |
| --- | --- | --- | --- | --- | --- | --- | --- |
| rs744877 | A | 1130  (66.0%) | 269  (68.6%) | 0.322 | 1130  (68.8%) | 0.082 | 0.940 |
|  | C | 582  (34.0%) | 123  (31.4%) |  | 512  (31.2%) |  |  |
|  | AA | 361  (42.2%) | 95  (48.5%) | 0.174 | 392  (47.8%) | 0.057 | 0.846 |
|  | AC | 408  (47.6%) | 79  (40.3%) |  | 346  (42.1%) |  |  |
|  | CC | 87  (10.2%) | 22  (11.2%) |  | 83  (10.1%) |  |  |
| rs3766526 | G | 1406  (82.1%) | 317  (80.9%) | 0.559 | 1319  (80.3%) | 0.183 | 0.809 |
|  | A | 306  (17.9%) | 75  (19.1%) |  | 323  (19.7%) |  |  |
|  | GG | 574  (67.1%) | 127  (64.8%) | 0.832 | 532  (64.8%) | 0.272 | 0.768 |
|  | GA | 258  (30.1%) | 63  (32.1%) |  | 255  (21.1%) |  |  |
|  | AA | 24  (2.8%) | 6  (3.1%) |  | 34  (4.1%) |  |  |

(# P value for AITD patients with family history versus Controls; & P value for AITD patients without family history versus Controls; * P value for AITD patients with family history versus AITD patients without family history. AITD, Autoimmune thyroid disease)

Supplementary table 5 Allele frequencies and genotype distribution of CD160 polymorphisms between GD patients and controls stratified by family history

| **SNP** | **Allele/**  **Genotypes** | **Controls (%)** | **GD with family history (%)** | **P^#^** | **GD without family history (%)** | **P^&^** | **P^*^** |
| --- | --- | --- | --- | --- | --- | --- | --- |
| rs744877 | A | 1130  (66.0%) | 191  (70.7%) | 0.125 | 694  (69.5%) | 0.059 | 0.703 |
|  | C | 582  (34.0%) | 79  (29.3%) |  | 304  (30.5%) |  |  |
|  | AA | 361  (42.2%) | 68  (50.4%) | 0.202 | 246  (49.3%) | 0.028 | 0.898 |
|  | AC | 408  (47.7%) | 55  (40.7%) |  | 202  (40.5%) |  |  |
|  | CC | 87  (10.1%) | 12  (8.9%) |  | 51  (10.2%) |  |  |
| rs3766526 | G | 1406  (82.1%) | 211  (78.2%) | 0.117 | 802  (80.4%) | 0.254 | 0.421 |
|  | A | 306  (17.9%) | 59  (21.8%) |  | 196  (19.6%) |  |  |
|  | GG | 574  (67.1%) | 81  (60.0%) | 0.269 | 326  (65.3%) | 0.212 | 0.371 |
|  | GA | 258  (30.1%) | 49  (36.3%) |  | 150  (30.1%) |  |  |
|  | AA | 24  (2.8%) | 5  (3.7%) |  | 23  (4.6%) |  |  |

(# P value for GD patients with family history versus Controls; & P value for GD patients without family history versus Controls; * P value for GD patients with family history versus GD patients without family history. GD, Graves' disease)

Supplementary table 6 Allele frequencies and genotype distribution of CD160 polymorphisms between HT patients and controls stratified by family history

| **SNP** | **Allele/**  **Genotypes** | **Controls (%)** | **HT with family history (%)** | **P^#^** | **HT without family history (%)** | **P^&^** | **P^*^** |
| --- | --- | --- | --- | --- | --- | --- | --- |
| rs744877 | A | 1130  (66.0%) | 78  (62.9%) | 0.641 | 436  (67.7%) | 0.437 | 0.417 |
|  | C | 582  (34.0%) | 44  (36.1%) |  | 208  (32.3%) |  |  |
|  | AA | 361  (42.2%) | 27  (44.3%) | 0.225 | 146  (45.3%) | 0.610 | 0.317 |
|  | AC | 408  (47.7%) | 24  (39.3%) |  | 144  (44.7%) |  |  |
|  | CC | 87  (10.1%) | 10  (16.4%) |  | 32  (10.0%) |  |  |
| rs3766526 | G | 1406  (82.1%) | 106  (86.9%) | 0.182 | 517  (80.3%) | 0.302 | 0.086 |
|  | A | 306  (17.9%) | 16  (13.1%) |  | 127  (19.7%) |  |  |
|  | GG | 574  (67.1%) | 46  (75.4%) | 0.394 | 206  (64.0%) | 0.579 | 0.215 |
|  | GA | 258  (30.1%) | 14  (23.0%) |  | 105  (32.6%) |  |  |
|  | AA | 24  (2.8%) | 1  (1.6%) |  | 11  (3.4%) |  |  |

(# P value for HT patients with family history versus Controls; & P value for HT patients without family history versus Controls; * P value for HT patients with family history versus HT patients without family history. HT, Hashimoto's thyroiditis)

Supplementary table 7 Allele frequencies and genotype distribution of CD160 polymorphisms between AITD patients and controls stratified by goiter

| SNP | Allele/  Genotypes | Controls (%) | Non-goiter (%) | **P^#^** | Goiter I (%) | **P^&^** | Goiter II (%) | **P^*^** | Goiter III (%) | **P^§^** |
| --- | --- | --- | --- | --- | --- | --- | --- | --- | --- | --- |
| rs744877 | A | 1130  (66.0%) | 388  (68.8%) | 0.223 | 347  (66.0%) | 0.988 | 597  (70.9%) | 0.013 | 67  (65.7%) | 0.947 |
|  | C | 582  (34.0%) | 176  (31.2%) |  | 179  (34.0%) |  | 245  (29.1%) |  | 35  (34.3%) |  |
|  | AA | 361  (42.2%) | 133  (47.2%) | 0.337 | 114  (43.4%) | 0.736 | 218  (51.8%) | 0.003 | 22  (43.1%) | 0.906 |
|  | AC | 408  (47.7%) | 122  (43.3%) |  | 119  (45.2%) |  | 161  (38.2%) |  | 23  (45.1%) |  |
|  | CC | 87  (10.1%) | 27  (9.5%) |  | 30  (11.4%) |  | 42  (10.0%) |  | 6  (11.8%) |  |
| rs3766526 | G | 1406  (82.1%) | 460  (81.6%) | 0.762 | 424  (80.6%) | 0.430 | 671  (79.7%) | 0.138 | 81  (79.4%) | 0.488 |
|  | A | 306  (17.9%) | 104  (18.4%) |  | 102  (19.4%) |  | 171  (20.3%) |  | 21  (20.6%) |  |
|  | GG | 574  (67.1%) | 189  (67.0%) | 0.634 | 169  (64.3%) | 0.703 | 268  (63.7%) | 0.260 | 33  (64.7%) | 0.454 |
|  | GA | 258  (30.1%) | 82  (29.1%) |  | 86  (32.7%) |  | 135  (32.1%) |  | 15  (29.4%) |  |
|  | AA | 24  (2.8%) | 11  (3.9%) |  | 8  (3.0%) |  | 18  (4.2%) |  | 3  (5.9%) |  |

(# P value for non goiter-AITD patients versus Controls; & P value for AITD patients with goiter I versus Controls; * P value for AITD patients with Goiter II versus controls; **^§^**P value for AITD patients with Goiter III versus controls; AITD, Autoimmune thyroid disease)
